# Degree-day models for predicting adult *Delia platura* (Diptera: Anthomyiidae) spring flight and first emergence in New York State

**DOI:** 10.1093/jee/toae148

**Published:** 2024-07-18

**Authors:** Paola Olaya-Arenas, Chloe Yi-Luo Cho, Daniel Olmstead, Anna DiPaola, Shea Crowther, Janice Degni, Jeff Miller, Aaron Gabriel, Mike Stanyard, Marion Zuefle, Jodi Letham, Katja Poveda

**Affiliations:** Faculty of Engineering, Design, and Applied Sciences, Universidad Icesi, Cali, Colombia; Department of Entomology, Cornell University, Ithaca, NY, USA; New York State Integrated Pest Management, Cornell University, Geneva, NY, USA; Department of Ecology and Evolutionary Biology, Cornell University, Ithaca, NY, USA; Department of Entomology, Cornell University, Ithaca, NY, USA; Cornell Cooperative Extension, Cornell University, Ithaca, NY, USA; Cornell Cooperative Extension, Cornell University, Ithaca, NY, USA; Cornell Cooperative Extension, Cornell University, Ithaca, NY, USA; Cornell Cooperative Extension, Cornell University, Ithaca, NY, USA; New York State Integrated Pest Management, Cornell University, Geneva, NY, USA; Cornell Cooperative Extension, Cornell University, Ithaca, NY, USA; Department of Entomology, Cornell University, Ithaca, NY, USA

**Keywords:** emergence, corn, degree-day model, New York State, predictive model

## Abstract

The seedcorn maggot, *Delia platura* (Meigen), is a pest affecting many crops, including corn. The early spring emergence of adults and belowground seed damage by maggots leave no room for rescue treatments during the short growing season in New York State. Degree-day (DD) models play a crucial role in predicting insect emergence and adult peak activity and are essential for effective pest management. The current *D. platura* DD model was launched on the Network for Environment and Weather Applications (NEWA) in 2022, using existing scientific literature from other North American regions. The NEWA model predicted adult *D. platura* first emergence at an average of 471 (39°F) DD in 2022. To gain an accurate and precise understanding of *D. platura* adult spring emergence and activity, we used interpolated temperature data to calculate the DD for each specific location where adults were captured in the field. DD calculations were performed using the average method, setting a biofix on January 1st and a base temperature of 39°F. In 2023, overwintering adults emerged at an average of 68 DD, and in 2022, adult activity was registered at an average of 282 DD. Accurately predicting the emergence of *D. platura* could contribute to informing integrated pest management strategies that incorporate timing and cultural practices over chemical solutions to protect crops and the environment.

## Introduction

*Delia platura* (Meigen) is a cosmopolitan detritivore and herbivore pest posing a risk to various crops, such as beans, peas, cereals, corn, and many crucifers (Throne 1980, Higley and Pedigo 1984a, Finch 1989, Hammond 1990, Tejeda-Reyes et al. 2022). In New York State (NYS), infestations by *D. platura* larvae can be observed in small numbers on newly planted crops throughout the growing season, resulting in detrimental effects on seed germination and growth of seedlings (Vea et al. 1975, Richards 2006). In severe cases, such damage can cause significant stand loss and reductions in crop yields requiring replanting of affected fields (Vea and Eckenrode 1976, Malone et al. 2004). To protect vulnerable crops during the critical stages of germination and seedling emergence, many growers employ insecticide seed treatments (Eckenrode et al. 1973, Montecinos et al. 1986, Salgado and Nault 2022).

As an alternative to seed treatments, predicting the first emergence in spring allows growers to implement the cultural practice of delaying planting dates. Employing early or late planting as a strategy against *D. platura* damage is effective if overwintering population emergence is accurately predicted, and weather conditions remain relatively stable. Insect phenology fluctuates annually due to weather conditions, resulting in earlier emergence during warmer years compared to colder ones (Herms 2004). Each insect species progresses through distinct life stages driven by specific heat accumulation requirements, estimated using degree-day (DD) values (Zalom et al. 1983, Murray 2008). *Delia platura* development requires temperatures between 39°F (3.9°C) and 84°F (29°C) (Higley and Pedigo 1984a). The accumulation of winter and early spring DD influences the timing of adult *D. platura* emergence, thereby impacting efforts to monitor and improve management practices.

DD models are crucial for integrated pest management (IPM), facilitating year-round pest activity monitoring and strategic control planning (Pruess 1983). The accuracy of DD relies on precise temperature measurements from nearby weather stations to mitigate microclimate variations impacting pest predictions at specific sites (Herms 2004). While studies report early adult *D. platura* emergence in garlic fields in Turkey (March 15) and onion fields in California (March 26), they lack information on the DD accumulations at which individuals were caught (Wilson et al. 2015, Erdogan 2019). The 2022 Network for Environment and Weather Applications (NEWA) model for NYS predicted first adult emergence at an average of 471 DD, using the average method, 39°F as a base temperature, and a biofix set on January 1st. However, the overwintered population has been reported to reach the peak emergence (50% individuals) at ≤360 DD (Barbercheck 2022, Dean and Hodgson 2023, Varenhorst 2023). The lack of precise information on DD at which first spring emergence occurs limits the use of DD as a cultural method for controlling *D. platura* in corn and other susceptible crops.

Knowing the DD at which adult *D. platura* emerges in the field plays a pivotal role in the successful implementation of IPM programs in NYS. It makes it possible to develop site-specific management strategies aligned with *D. platura* phenology in both space and time. This research focuses on enhancing the current *D. platura* adult emergence model developed by the New York State Integrated Pest Management Program (NYSIPM) for use on the NEWA agricultural risk assessment platform (https://newa.cornell.edu). To achieve this objective, *D. platura* adults emerging in early spring were caught with sticky cards at 22 sites between April 19 and 30, in 2022 and at 4 sites between March 7 and April 6 in 2023. For each site, the accumulated DD was calculated using the average method from January 1st until the last date that sticky cards were at each field. Identifying adult *D. platura* first emergence during the spring will help to develop an IPM strategy for a critical period characterized by heightened susceptibility of corn seeds, seedlings, and other crops to damage caused by this pest.

## Materials and Methods

### Monitoring *D. platura* Flight Activity

**Fig. 1.**
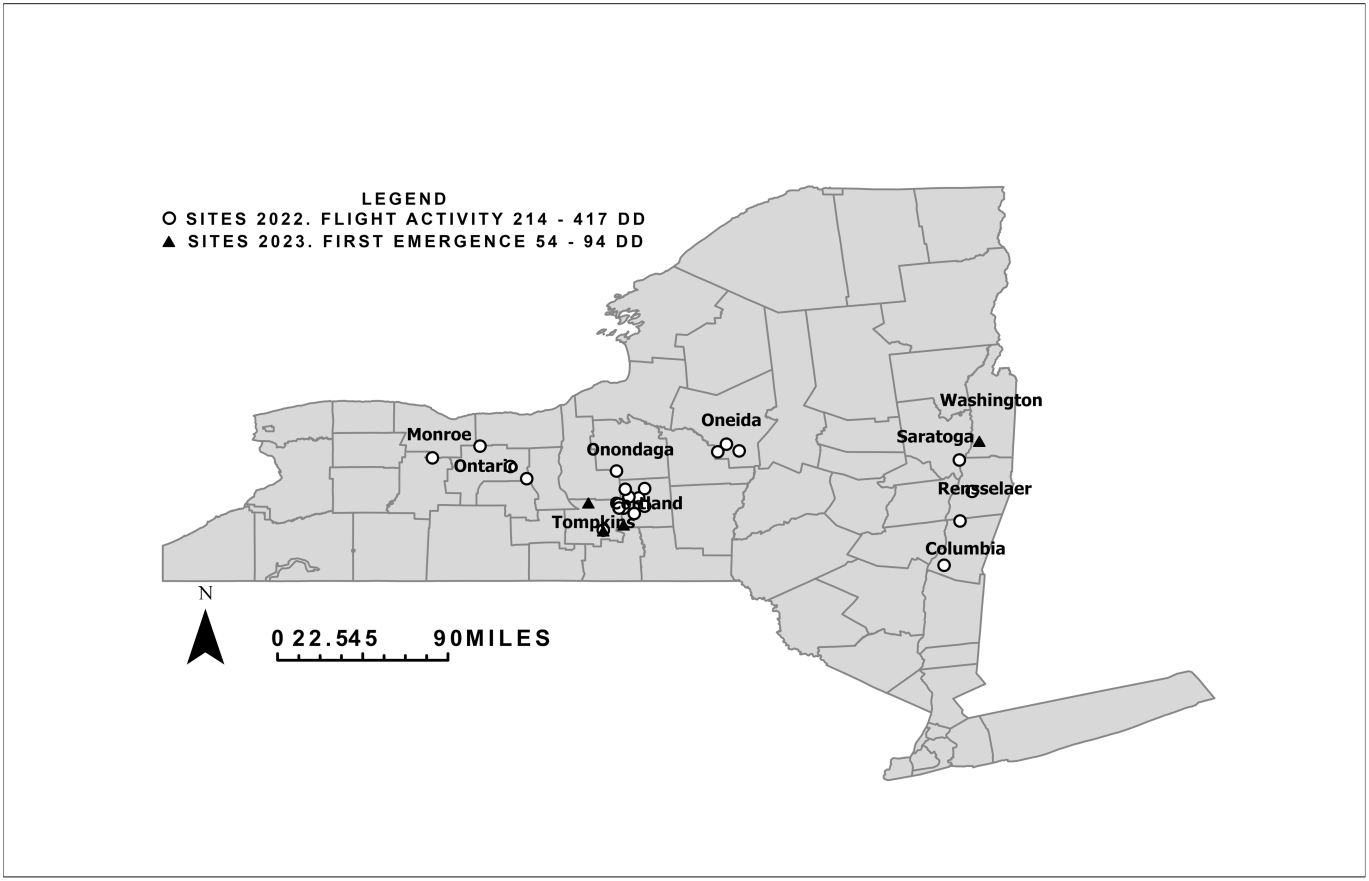
Twenty-six fields sampled in 10 counties in NYS in 2022 (white circles) and 2023 (black triangles). The site symbols for one location overlap because it was sampled in 2022 and 2023.

Data were collected in 26 corn fields across NYS (Fig. 1). Twenty-two fields were sampled in 2022, between April 19 and 30, 2022, and 4 fields were sampled in 2023, between March 7 and April 6, 2023. At the edge of each field, 2 blue sticky cards (16.5 × 10.2 cm, Solida Distributions, Quebec, Canada), with adhesive on both sides, were attached to 2 wooden stakes (2.5 × 2.5 cm and 9.4 cm) with a spring clamp at 22 cm above the ground. The wooden stakes were separated by 30 m. The second sticky card was a duplicate in case the other one was damaged or lost. The cards were collected weekly. For both years, adult *D. platura* were sexed and counted on both sides on one sticky card. *D. platura* adults were identified in the lab using a *Delia* taxonomic key (Savage et al. 2016).

### Temperature and Precipitation Data

Daily minimum and maximum temperature values were retrieved for each location from The Regional Climate Center Applied Climate Information System platform (RCC-ACIS), using latitude and longitude coordinates to access 4-km resolution PRISM data (https://www.rcc-acis.org/docs_webservices.html#title24).

### Degree-Days

The average method was employed to calculate the accumulated DD for each site. The average method assumes that development occurs when minimum temperatures surpass the development threshold (Pruess 1983). The *D. platura* lower base development threshold is 39°F. For each location, the daily minimum and maximum temperatures were used to calculate heat units and the accumulated DD from January 1st until the calendar date at which adult *D. platura* were caught in the field. Calculations were done in JMP 13.0 (SAS Institute, Cary, NC).

The average method formula used is:


$$\begin{array}{l} \frac{\left(Maximum\;temperature\;+\;Mimumum\;temperature\right)\;\;\;\;\;}{2}\;-39^{\circ }\;F \end{array}$$

## Results

### Monitoring *D. platura* Flight Activity

**Fig. 2.**
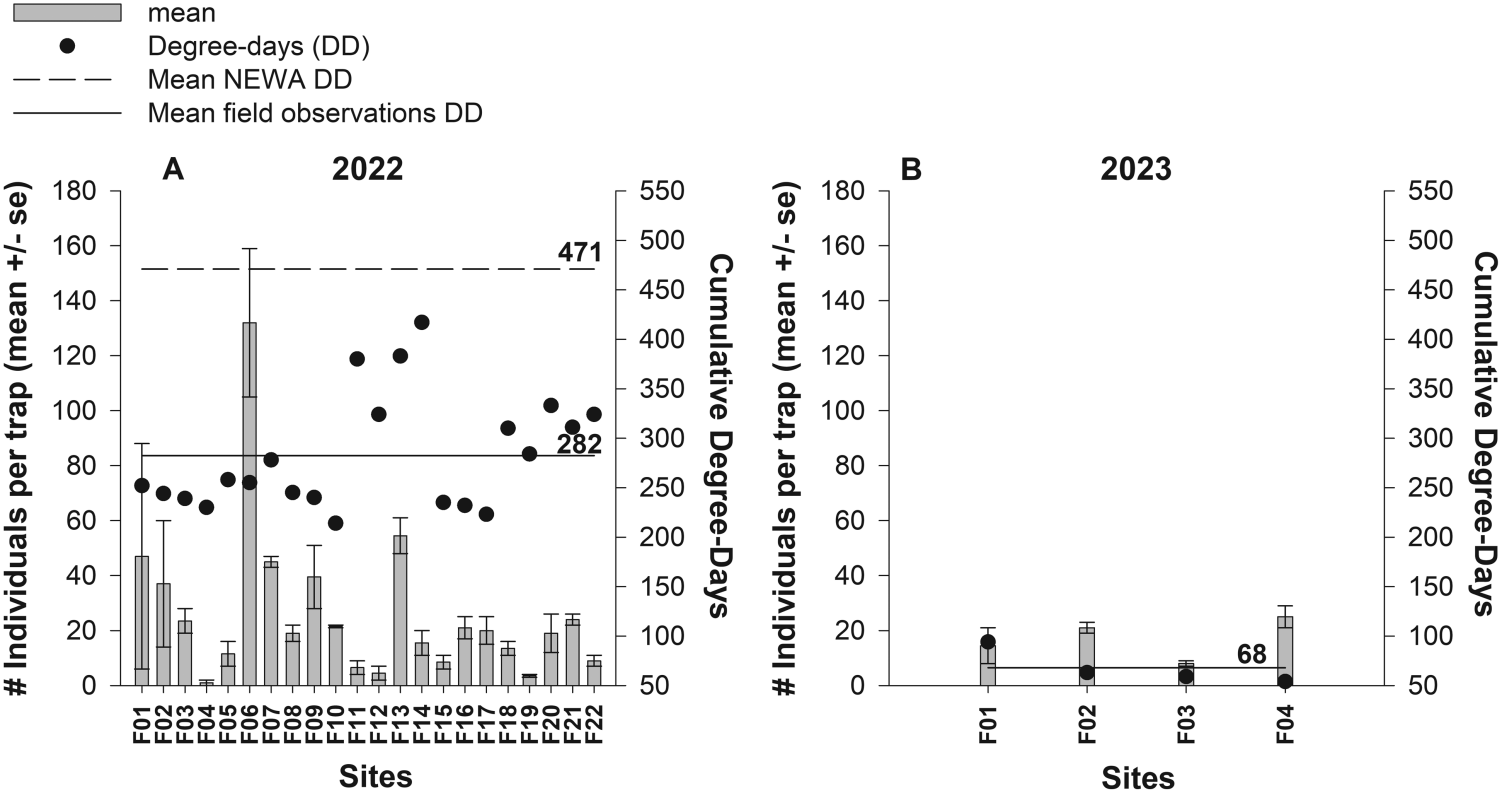
Adult *D. platura* counts (mean ± SE) at 22 and 4 corn fields in New York State in 2022 (A) and 2023 (B). The solid black circles represent the accumulated degree-days at which individuals were caught at each site. The horizontal black dashed line shows the average degree-days predicted by the NEWA model and the black solid lines show the average degree-days calculated using the field observations for each year.

**Fig. 3.**
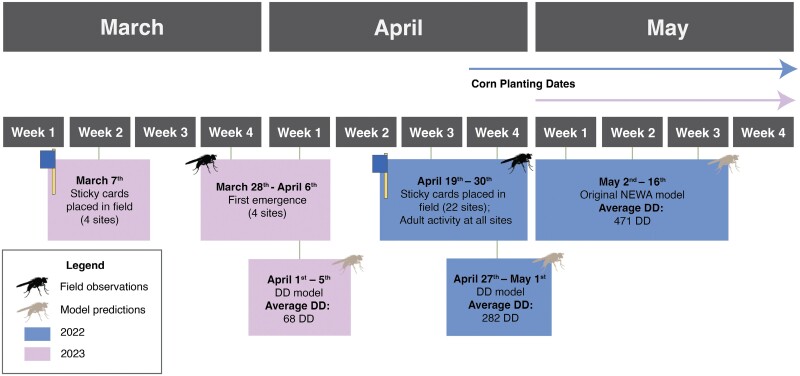
The timing of field observations and model predictions in 2022 and 2023. The graph includes the mean accumulated DD for early spring activity (2022) and first emergence (2023). Additionally, the graph displays the dates on which corn was planted by growers in 2022 (upper line) and 2023 (lower line).

The mean *D. platura* adults (flies caught/trap) varied across all locations and 2 years (Fig. 2A and B). In 2022, adults were trapped at all locations sampled between April 19 and 30, 2022, making it impossible to determine first emergence. In 2023, the first emergence of *D. platura* adults could be determined between March 21 and 31. This first emergence was 5–7 weeks earlier than the initial NEWA predictions (Fig. 3).

### Degree-Days

On a DD scale, NEWA predicts the peak adult emergence of *D. platura* in the field at an average of 471 DD. In 2022, adults were observed in the field at an average of 282 DD, with a range from 214 to 417 DD (Fig. 2A). In 2023, the first emergence of adults was observed at an average of 67 DD, with a range from 54 to 94 DD (Fig. 2B).

## Discussion

Our aim was to determine the accumulated DD at which *D. platura* adults are present in the field in early spring, in NYS. In 2022, traps were placed on April 19, capturing flies on all cards by April 30, suggesting that adults were active in the field before traps were set up. This is an unexpected result, but dates are not far away from the reported emergence of *D. platura* between April 12 and May 1 in Ontario (Canada) between 1951 and 1958 (Miller and McClanahan 1960), and middle to late April in Iowa (Higley and Pedigo 1984a) and New York (Throne and Eckenrode 1985). In 2023, cards were placed on March 7, and the first emerged adults were trapped between March 28 and April 6. The earlier adult flight activity and emergence DD, compared to the original NEWA model prediction, indicate that *D. platura* is active in the field much earlier than previously reported.

Warmer temperatures might have contributed to the earlier emergence of *D. platura*. The year 2023 was the warmest year in the 174-yr observational record maintained by the World Meteorological Organization (WMO). Warmer temperatures accelerate the accumulation of DD, leading to faster development of insects and earlier emergence, as demonstrated for *D. platura* by Harukawa et al. (1934) and Sanborn et al. (1982). The effect of warming temperatures on *D. platura* emergence has been considered in Iowa, where overwintered *D. platura* emerged on February 27 (Higley and Pedigo 1984a), and in California, where a change of 3 °C in the average daily air temperature caused an earlier emergence in 2013 (March 26 to April 6) compared to 2011 (May 3 to 12) (Wilson et al. 2015). However, warmer air temperatures alone do not explain the large differences in DD between previous models and our findings. The current models have a biofix of January 1st and are based on air temperatures. Our current data make us reconsider the usefulness of these parameters for at least 2 reasons: (i) the temperature in the soil is much higher than the air temperature, and given the milder winters, it is possible that we are accumulating growing DD in the soil that we are not registering when using air temperature and (ii) the warmer days during the fall (before the start of the biofix date set on January 1st) could also be contributing to the quicker development of *D. platura*. We are currently working on obtaining more data to discern the underlying difference in previous predictive models and current conditions to provide more accurate recommendations in the future.

Determining the first emergence range across NYS and linking the number of individuals caught in the field to seed damage and crop yield reduction is crucial. Studies report the accumulated DD (360 DD) at which 50% of individuals are caught (Sanborn et al. 1982, Silver et al. 2018), associating this with a female oviposition period (Barbercheck 2022, Dean and Hodgson 2023, Varenhorst 2023). However, details about the exact number of individuals and the extent of damage to seeds and crops remain unknown. We need to continue gathering information about the cumulative DD for adult emergence and follow the growth of the population to find the adult peaks causing significant damage. This will help provide effective monitoring and management strategies to control *D. platura*.

Revised criteria for detecting the first adult *D. platura* will be integrated into the current NEWA model, with ongoing validation efforts expected to refine the model further. With these updates, the NEWA model will offer a more accurate and precise measurement of *D. platura* adult phenology throughout the growing season, serving as a crucial tool for growers, crop consultants, and regulatory staff. It will function as an early warning and predictive mechanism to monitor early spring population emergence.

Considering additional factors that influence maggot infestation, seed injury, stand loss, and yield reduction can complement DD models. For instance, temperature, solar radiation, and nutrition play roles in the development of *D. platura* and its reproductive fitness (Sanborn et al. 1982, Funderburk et al. 1984, Higley and Pedigo 1984b). Future research should prioritize enhancing our understanding of larval development and seed damage risk during spring, particularly about soil warming temperature variations across different soil types.
